# Study of Potential Anti-Inflammatory Effects of Red Wine Extract and Resveratrol through a Modulation of Interleukin-1-Beta in Macrophages

**DOI:** 10.3390/nu10121856

**Published:** 2018-12-01

**Authors:** Pauline Chalons, Souheila Amor, Flavie Courtaut, Emma Cantos-Villar, Tristan Richard, Cyril Auger, Philippe Chabert, Valérie Schni-Kerth, Virginie Aires, Dominique Delmas

**Affiliations:** 1 Université de Bourgogne, F-21000 Dijon, France; paulinechalons@orange.fr (P.C.); souheila.amor@u-bourgogne.fr (S.A.); flaviecourtaut@gmail.com (F.C.); virginie.aires02@u-bourgogne.fr (V.A.); 2 INSERM Research Center U1231–Cancer and Adaptative Immune Response Team–Bioactive Molecules and Health research group, F-21000 Dijon, France; 3 Intituto de Investigación y Formación Agraria y Pesquera (IFAPA) Rancho de La Merced, Ctra. Trebujena, 11.471 Jerez de la Frontera (Cadiz), Spain; flaviecourtaut@gmail.com; 4 Université de Bordeaux, Unité de Recherche Œnologie, EA 4577, USC 1366 INRA, Equipe Molécules d’Intérêt Biologique-ISVV, F-33882 Villenave d’Ornon, France; tristan.richard@u-bordeaux.fr; 5 UMR 1260 INSERM Nanomédecine Régénérative, Université de Strasbourg, F-67401 Illkirch, France; cyril.auger@unistra.fr (C.A.); valerie.schini-kerth@unistra.fr (V.S.-K.); 6 UMR CNRS 7021-Laboratoire de Bioimagerie et Pathologies-Université de Strasbourg, F-67401 Illkirch-Graffenstaden, France; pchabert@unistra.fr

**Keywords:** red wine extract, polyphenols, resveratrol, inflammation, interleukins

## Abstract

Inflammation has been described as an initiator event of major diseases with significant impacts in terms of public health including in cardiovascular disease, autoimmune disorders, eye diseases, age-related diseases, and the occurrence of cancers. A preventive action to reduce the key processes leading to inflammation could be an advantageous approach to reducing these associated pathologies. Many studies have reported the value of polyphenols such as resveratrol in counteracting pro-inflammatory cytokines. We have previously shown the potential of red wine extract (RWE) and the value of its qualitative and quantitative polyphenolic composition to prevent the carcinogenesis process. In this study, we addressed a new effect of RWE in inflammation through a modulation of IL-1β secretion and the NLRP3 inflammasome pathway. NLRP3 inflammasome requires two signals, priming to increase the synthesis of NLRP3 and pro-IL-1β proteins and activation, which activates NLRP3. Inflammasome formation is triggered by a range of substances such as lipopolysaccharide (LPS). Using two different macrophages, one of which does not express the adaptor protein ASC (apoptosis-associated speck-like protein containing a CARD), which is essential to form active inflammasome complexes that produce IL-1β, we show that RWE decreases IL-1 β secretion and gene expression whatever line is used. Moreover, this strong reduction of pro-inflammatory IL-1β is associated with a decrease of NLRP3 and, in J774A, ASC protein expression, which depends on the choice of activator ATP or nigericin.

## 1. Introduction

For several years, numerous epidemiological studies have maintained that a moderate consumption of wine lowered the risks of mortality, in particular due to coronary diseases, compared to the risk observed with wine abstinence. [1,2]. In France, as compared with other western countries with a fat-containing-diet, the strikingly low incidence of coronary heart diseases is partly attributed to the moderate consumption of red wine [3]. In our previous studies, we have shown an improvement of blood parameters with the decrease in total cholesterol and LDL and the increase in erythrocyte membrane fluidity and antioxidant status on a group of selected post-myocardial infarct patients receiving 250 mL/day of red wine over 2 weeks in comparison to patients receiving water [4]. These cardiovascular benefit effects are commonly attributed to red wine’s rich content in polyphenols, particularly resveratrol, as an important source of antioxidants [5]. Interestingly, other epidemiological studies reveal that micronutrients such as resveratrol could protect against cancers [1]. However, resveratrol does not seem to be the only bioactive compound present in wine, which contains numerous other polyphenols [6]. Indeed, some studies have highlighted that other polyphenols of red wine such as quercetin [7], catechin [8], and gallic acid [9] could present potential chemopreventive properties. Various case–control studies have shown that moderate red wine consumption exerts a protective effect on colorectal cancer in both men and women [10,11]. Moreover, other case–control studies have studied the association between wine and the Mediterranean diet, showing a lower risk of colon cancer compared to other diets [12,13,14]. Nevertheless, one study did not find an inverse association between moderate red wine intake and the risk of colorectal cancer [15] or breast cancer [16]. This controversy may result from the amount and quality of polyphenols present in red wine. Indeed, red wine contains a range of biologically active polyphenols, including phenolic acids, trihydroxystilbenes, and flavonoids. In previous studies, we have shown both that a mixture of polyphenols extracted from vine shoots presents a better antiproliferative activity on colon cancer cells than resveratrol alone due to a synergism between polyphenols [17] and that the quantity and quality of the polyphenols present in the wine also played an important role. We showed that the wine making procedure can increase the quantities of certain polyphenols, especially lengthening the maceration time, which results in an improvement of the biological effects of these red wine extracts, which are then able to slow down the formation of aberrant crypt foci (ACF) [18] and angiogenesis [19]. But very interestingly, some polyphenols do not act in a synergistic manner but rather in an additive manner and in some cases they have opposite effects. These data raise the question of the crucial role of the polyphenol composition of wine where an imbalance between polyphenolic species may increase or conversely reduce their effects. The presence of catechin reduces the synergism effect between resveratrol and quercetin, which could explain the discrepancy between some studies showing a reduction of the risk of colon cancer with moderate red wine consumption in patient [10,11] or in animal models [19,20] while others showed no effect [15,21].

To conduct our previous studies, we investigated the potential effects of a red wine extract (RWE) on a fundamental biological element in the occurrence of various pathologies, in particular cancer, namely inflammation and more particularly the production of IL-1β.

Inflammation can be initially defined as a set of physiological defense reactions put in place by the body and more particularly by the cells of innate immunity. These cells play a role in first-line defense following various traumas caused by infectious agents, chemical substances, physical agents or even post-traumatic tissue lesions. In contrast, inflammation has been described as an initiator event of major diseases with a significant impact in terms of public health such as cardiovascular diseases, autoimmune diseases, eye diseases, age-related diseases, and more particularly neurodegenerative diseases and the occurrence and development of cancers. More particularly, low-level inflammation seems crucial in sustaining these processes. Among polyphenols and flavonoids present in the human diet, resveratrol seems to inhibit NLRP3 inflammasome-derived IL-1β secretion in the J774A.1 murine macrophage cell line and pyroptosis [22]. This point is particularly important because there is a link between activation/controls of NLRP3 and various pathologies, i.e., atherosclerosis [23], Alzheimer’s disease [24], cancers [25], and renal disease [26]. Despite numerous studies showing the impact of polyphenols alone on the inflammation process, there are still very few data on the effect of complex mixtures of polyphenols such as red wine extract (RWE). Some studies have pointed out the potential anti-inflammatory role of RWE in colon cancer cells through a pathway involving both an activation of the nuclear factor-erythroid 2-related factor-2 (Nrf2) pathway and an inhibition of the Janus kinase/signal transducer and activator of transcription (JAK/STAT) pathway [27] or through a disruption of colonic NADPH-oxidase NOX1 activation [28]. These effects lead to a decrease of the pro-inflammatory cytokines Il-6 and IL-8. In view of the importance of the anti-inflammatory potential of RWE, it is therefore essential to better characterize the effects of a polyphenol-enriched extract such as RWE on one of the essential pro-inflammatory cytokines, namely IL-1β, on the mechanisms conducive to its secretion. In this study, we show that RWE was able to strongly decrease IL-1β secretion through a modulation of the expression of key proteins involved in the inflammasome complex, i.e., NLRP3 and apoptosis-associated speck-like protein containing a CARD (ASC). Moreover, we highlight that RWE was able to affect the priming signal and the activating signal leading to inflammasome activation in macrophages.

## 2. Materials and Methods

### 2.1. Cells Culture

The murine macrophage cell line J774A.1 and Raw 264.7 were obtained from the European Collection of Authenticated Cell Cultures (ECACC, Salisbury, UK) and from the American Type Culture Collection (ATCC, Manassas, VA, USA), respectively. Raw 264.7 cells were cultured in RPMI 1640 medium supplemented with 10% FBS and 10,000 U/mL penicillin, 10 mg/mL streptomycin, 25 μg/mL amphotericin at 37 °C in a 5% CO_2_ incubator; therefore, J774A.1 cells were cultured in DMEM high-glucose medium supplemented with 10% fetal bovine serum (FBS), with 2 mM L-glutamine and 10,000 U/mL penicillin, 10 mg/mL streptomycin, 25 μg/mL amphotericin at 37 °C in a 5% CO_2_ incubator. Cells were subcultured twice weekly using standard protocols.

### 2.2. Preparation of Red Wine Extract

Red wine extract (RWE) was obtained from French red wine, Santenay 1er cru Les Gravières 2012 (EARL Capuano-Ferreri Santenay, Côte-d’Or, France) selected by BIVB (Bureau Interprofessionnel des Vins de Bourgogne, Beaune, France) and provided by CTIVV (Centre Technique Interprofessionnel de la Vigne et du Vin, Beaune, France). Red wine extract dry powder was prepared and analyzed as previously described [19]. Briefly, phenolic compounds were adsorbed on a preparative column, then alcohol desorbed. The alcoholic eluent was gently evaporated, and the concentrated residue was lyophilized and finely sprayed to obtain the phenolic extract dry powder. Briefly, the alcoholic eluent and water were gently evaporated using a rotary evaporator set, and the concentrated residue was deposited on the column (Diainon^®^ HP-20, Supelco, Germany). The reservoir was filled with distilled water and the flow was adjusted to about 20 drops/min. The polyphenol fraction was eluted with an ethanol-0.1% glacial acetic acid solution (flow adjusted to 40 drops/min). The individual eluent fractions were collected and concentrated to dryness using a rotary evaporator set. One liter of red wine produced 104 g of phenolic extract, which contained 5.04 mg/g of total phenolic compounds expressed as gallic acid equivalent.

### 2.3. HPLC Analysis

The phenolic composition of the wine samples was determined by HPLC analysis following the methods developed by Guererro et al. (2009) [29]. Briefly, HPLC analysis was performed on a HPLC system equipped with a diode array detector, a fluorescence detector and a C18 column (5 μm, 250 mm × 4.6 mm) for compound separation. For anthocyanins and flavonols, the mobile phase consisted of water containing 5% formic acid (v/v) and methanol in various proportions at a flow rate of 1 mL/min. Anthocyanins were quantified at 520 nm as malvidin-3-glucoside and flavonols at 360 nm as quercetin-3-rutinoside. Flavan-3-ols and phenolic acids were analyzed using a gradient containing water with 2% acetic acid (v/v) and methanol–water–acetic acid (90:8:2, v/v/v). Phenolic acids were quantified at 320 nm as caffeic acid, flavan-3-ols as catechin using the fluorescence signal (excitation wavelength, 290 nm; emission wavelength, 320 nm) and stilbenes as resveratrol (excitation wavelength, 330 nm; emission wavelength, 320 nm).

### 2.4. Reagents

Resveratrol (RSV), LPS (from *Escherichia coli* 0111: B4, L3024), adenosine 5-triphosphate disodium salt solution (A6559) and nigericin sodium salt (N7143) were purchased from Sigma-Aldrich (St. Louis, MO, USA).

### 2.5. Experimental Protocol

Cells were seeded at the density of 10,000 cells/cm² and allowed to recover for 24 h. As usually done, after 24 h, to initiate NLRP3 inflammasome priming, cells were pretreated or not with 100 μg/mL RWE or 60 μM RSV for 30 min and then primed with 1 μg/mL LPS (5.5 h) and finally exposed for an additional 30 min to 10 μM nigericin or 5 mM ATP [22]. For the activation signal analyses, cells were first primed with 1 μg/mL LPS (5.5 h), then treated or not with 100 μg/mL RWE or 60 μM RSV for 30 min and finally with 10 μM of nigericin or 5 mM ATP, as previously described by Chang et al. [22].

Priming and activation are annotated RWE/RSV->LPS->Nig/ATP and LPS->RWE/RSV->Nig/ATP, respectively.

### 2.6. Cell Proliferation Assay

Cells were seeded in 96-well flat-bottomed microplates and incubated for 24 h. The medium was then removed and replaced with fresh medium containing the RWE or RSV to be tested at increasing concentrations (from 1.9 to 250 μg/mL) at 37 °C for 24 h. Each treatment was performed in sixplicate (in three independent experiments). The activity of compounds was determined using a solution of crystal violet (Sigma-Aldrich, St. Louis, MO, USA). Absorbance at 540 nm was measured by Biochrom Asys UVM 340. IC_50_ (i.e., the half maximum inhibitory concentration representing the concentration of a substance required for 50% in vitro inhibition) values were calculated using GraphPad 6.0 Prism software (GraphPad Software, La Jolla, San Diego, CA, USA).

### 2.7. Western Blotting

Cells were treated according to the experimental protocol described above, then were harvested for Western blot analysis in RIPA buffer (RadioImmunoPrecipitation Assay buffer; 150 mM sodium chloride, 50 mM Tris-HCl, 0.1% sodium dodecyl sulfate, 1% NP40, 0.5% sodium deoxycholate) supplemented with protease inhibitors such as phosphatase inhibitor cocktail (100 μM, Sigma-Aldrich, St. Louis, MO, USA) and an anti-protease (1x, Roche). The protein concentration of each lysate was determined in a 96-well plate against BSA standards in PBS (range, 0–12 μg), applying the QuantiPro™ BCA Assay Kit (Sigma-Aldrich, St. Louis, MO, USA), and the total amount of proteins per well was calculated. Samples were adjusted into Laemmli gel-loading buffer (50 mM Tris-HCl, pH 6.8, 5% 2-mercaptoethanol, 2% sodium dodecyl sulfate, 0.1% bromphenol blue, 10% glycerol) and then heated for 5 min at 95 °C prior to separation. Denatured proteins were separated by SDS-PAGE and transferred to nitrocellulose membranes (Amershan, GE, Velizy-Villacoublay, France). Membranes were blocked by incubation with skimmed milk (in TBS-Tween 20 0.5%) 1 h at room temperature. The membranes were incubated with the respective primary antibody: NLRP3 (clone cryo2, Adipogen^®^, Liestal, Switzerland), Asc (clone AL177, Adipogen^®^, Liestal, Switzerland) overnight at 4 °C according to the manufacturer’s recommendations. Afterwards, the membranes were incubated with HRP-conjugated secondary antibody, anti-rabbit and anti-mouse for ASC and NRLP3 (Jackson Immunoresearch Laboratory, Cambridgeshire, UK), respectively, at room temperature for 1 h and developed using the ECL reagents (Supersignal West Femto maximum sensitivity substrate, ThermoFisher Scientific, France). Antibody against housekeeping proteins such as β-actin was used as the loading control (clone AC-15, Sigma-Aldrich, St. Louis, MO, USA). Digital chemiluminescence images were captured and analyzed using the ChemiDocTM XRS + imaging system (BioRad, Marnes-la-Coquette, France). Image processing and analyses were carried out using Image Lab 5.2.1 build 11 Bio-Rad software (Berkeley, CA, USA).

### 2.8. Imaging

Cells were seeded on chambered coverglass coated with poly-L-lysin and allowed to recover. Cells were treated according to the experimental protocol described above. After the treatment, cells were fixed and permeabilized with 4% PAF for 10 min at 4 °C and then incubated with a blocking buffer (PBS1X-0.2% saponin-3% BSA) for 20 min at room temperature. The blocking buffer was eliminated, and the diluted antibodies were applied. Cells were incubated overnight at 4 °C in a humid light-tight box. Cells were rinsed thrice with PBS1X (5 min each), then incubated with the fluorochrome-conjugated secondary antibodies (Alexa488 and Alexa568 for NRLP3 and ASC, respectively) diluted as indicated on an antibody datasheet for 1 h at room temperature in a humid light-tight box. Cells were then washed thrice with PBS1X (5 min each time) then mounted with mounting medium with DAPI (ProLong^®^ antifade with Dapi, Life Technologies, ThermoFisher Scientific, Strasbourg, France). Confocal imaging was performed using a confocal laser-scanning microscope (Axio Imager M2, Zeiss) coupled with an Apotome.2 with a ×40 objective lens, and Zen software (Carl Zeiss Microscopy GmbH, Germany). The samples were excited using internal microscope lasers and emission intensity was recorded at the appropriate emission wavelength. Image processing and analyses were carried out using ImageJ software. Negative controls were treated with the fluorochrome-conjugated secondary antibodies alone.

### 2.9. IL-1β Secretion

Cells were treated according to the experimental protocol described above. Then IL-1β in the supernatant was measured using ELISA (Enzyme-Linked Immunosorbent Assay) for Mouse IL-1-beta^®^/IL-1F2 DuoSet^®^ ELISA (R&D Systems, Minneapolis, MN, USA) according to the manufacturer’s protocol.

### 2.10. Statistical Analysis

Results are shown as means ± SD for triplicate assay samples (otherwise mentioned), reproduced independently at least three times. Statistical analysis of data was carried out with Prism GraphPad 6.0 Prism Software (GraphPad Software, Inc. La Jolla, San Diego, CA, USA). The significance of the differences between mean values was determined by a one-way ANOVA with Holm-Sidak correction. *p*-values < 0.05 were considered significant (* *p* < 0.05, ** *p* < 0.01 and *** *p* < 0.001).

## 3. Results

### 3.1. RWE Decreases IL-1β Secretion and NLRP3 Expression in Murine Macrophages without Toxicity

**Figure 1:**
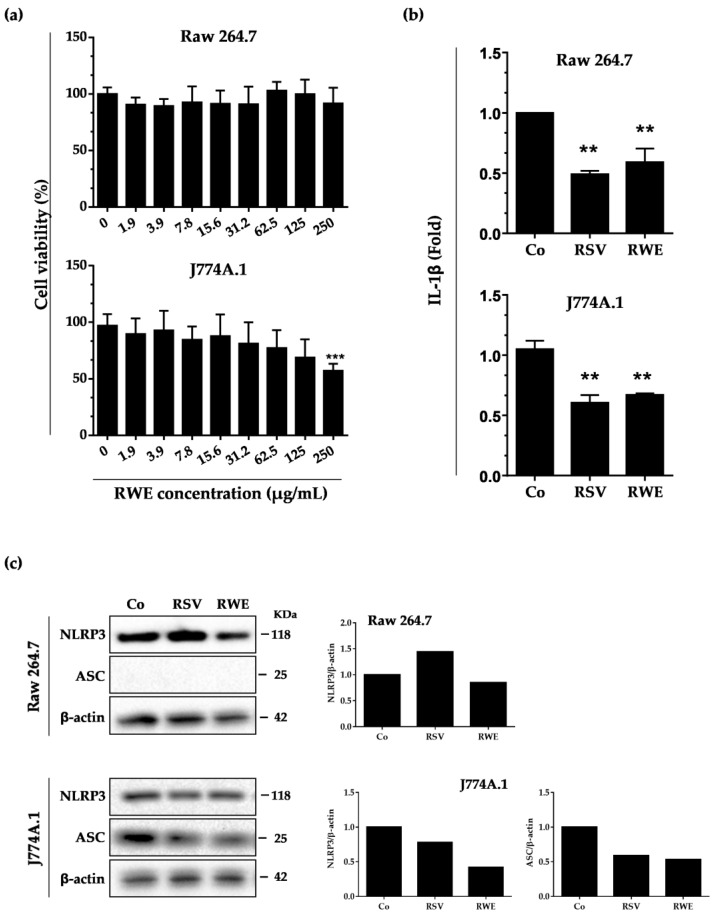
RWE and Resveratrol (RSV) decrease IL-1β secretion in resting conditions through the modulation of the protein expression levels of NLRP3 inflammasome components. (**a**) Cell viability of Raw 264.7 and J774A.1 was determined by crystal violet staining after 24 h of treatment, with concentration ranges of RWE (starting concentration 250 μg/mL, 1:2 serial dilutions). Data are expressed as mean percentages ± s.d. of three independent experiments. *p*-values were determined by a one-way ANOVA with Holm-Sidak correction. *** *p* < 0.001. (**b**) Secreted IL-1β in supernatants was analyzed by ELISA after 6 h of treatment with vehicle (DMSO, Co), RWE (100 μg/mL), or RSV (60 μM). Data are expressed as fold changes in secreted IL-1β levels as compared to vehicle-treated cells (Co) and are mean ± s.d. of three independent experiments. *p*-values were determined by the multiple Student *t* test. ** *p* < 0.01 (**c**) Western blot analysis of NLRP3 and ASC protein expression in Raw 264.7 and J774A.1 macrophages after 6.5 h of treatment with vehicle (DMSO, Co), RWE (100 μg/mL) or RSV (positive control). Left panels: representative blots from three independent experiments are shown. β-actin was used as loading control. Right panels: densitometry quantifications of representative blots. Plotted data are fold changes as compared to vehicle-treated cells (Co).

The composition of wine is a complex and unique combination due to the various factors such as the vine, the climate, the country and the year, and varies between white and red wines. The amount of polyphenols in wine, although varying greatly, is estimated to be around 190–290 mg/L in white wines and 900–2500 mg/L in red wines. We previously demonstrated that the qualitative and quantitative composition of a wine in bioactive molecules such as polyphenolic compounds is crucial in the biological effects that can be observed and that they are antagonistic or synergistic [18]. Also, we first evaluated the polyphenolic composition of our red wine extract, which contains significant amounts of anthocyanidins, catechins, and a polyphenol emblematic of the vine and wine, resveratrol, which in our experience will serve as a reference compound (Table 1). As compared to our previous studies, it appears that this polyphenolic extract of red wine is richer in caffeic acid and resveratrol [18]. To specify the potential role of RWE in inflammation, we first studied its effects on classical cellular models such as macrophages. To do this, we chose to use two murine macrophage lines, Raw264.7 and J774A.1, derived, respectively, from ascites of a male mouse bearing a tumor induced by murine Abelson leukemia virus and the blood of a tumor-bearing Balb/c female. As we have shown in previous studies that polyphenolic extracts and RWE induce toxicities in various cell lines, in particular against cancer cells [18,19] by disrupting their proliferation, we first evaluated the RWE toxicity (Figure 1a). We observe that after 24 h of treatment with increasing concentrations of RWE, polyphenolic extract has no impact on the cellular viability of Raw264.7 macrophages despite increasing concentrations of up to 250 μg/mL. However, in J774A.1 cells, RWE at 250 μg/mL reduced the number of viable cells by nearly 50%. In the following experiments, RWE was therefore used at 100 μg/mL in both cell lines. This concentration was chosen both because it is noncytotoxic on macrophages and because it significantly inhibited tumor growth and the formation of adenomatous polyps, as we have shown in several in vivo models [18,19]. The experiments were conducted in comparison with the RSV at a concentration of 60 μM, a concentration that is also noncytotoxic on macrophages and for which recent studies have shown an effect on NLRP3 [22].

Inflammation results from an increased production of pro-inflammatory cytokines. Among them, IL-1β plays a decisive role and its high secretion is responsible for the development of both chronic inflammation and an acute inflammatory response and its maintenance over time. RWE at 100 μg/mL is able to strongly decrease the basal secretion of IL-1β in the two macrophage types in a similar manner of resveratrol (RSV) at 60 μM after 30 min of treatment (Figure 1b). Since IL-1β production results mainly from the activation of NLRP3 inflammasome, we then studied the impact of a treatment with RWE. It appeared that RWE was able to slightly decrease NLRP3 protein expression after 6 h of treatment with 100 μg/mL in Raw264.7 (Figure 1c). Very interestingly, in J774.1, which is the only one of the two lines expressing the apoptosis-associated, speck-like protein containing a CARD domain (ASC), which plays a primordial role in inflammasome formation, RWE treatment strongly decreases ASC protein expression as compared to the control (Figure 1c).

### 3.2. RWE Prevents Priming of NLRP3 Inflammasome from LPS in Macrophages

**Figure 2:**
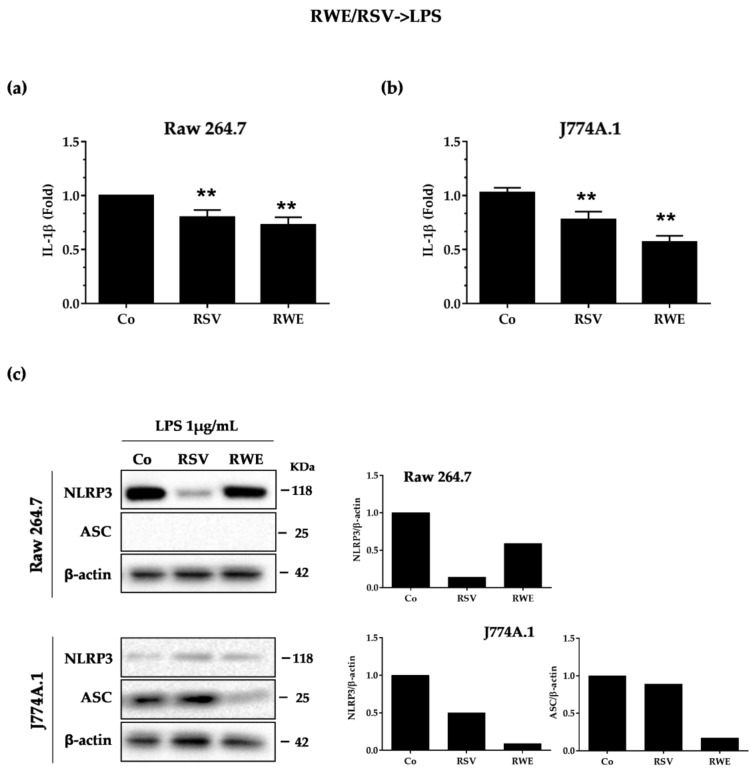
RWE and RSV decrease IL-1β secretion in lipopolysaccharide (LPS)-mediated NLRP3 inflammasome priming conditions. Raw 264.7 (**a**) and J774A.1 (**b**) macrophages were pretreated or not with RWE (100 μg/mL) or RSV (60 μM) for 30 min, prior to NLRP3 inflammasome priming with 1 μg/mL of LPS for 5.5 h. Secreted IL-1β in cell supernatants was analyzed by ELISA at the end of the treatments. Data are expressed as fold changes in secreted IL-1β levels as compared to cells treated with LPS alone (Co) and are mean ± s.d. of three independent experiments. *p*-values were determined by the multiple Student *t* test. ** *p* < 0.01 (**c**) Western-blot analysis of NLRP3 and ASC protein expression in Raw 264.7 and J774A.1 macrophages after cell treatments as described above. Left panels: representative blots from three independent experiments are shown. β-actin was used as loading control. Right panels: densitometry quantifications of representative blots. Plotted data are fold changes as compared to LPS-treated cells (Co).

Conventionally, activation of the NLRP3 inflammasome requires two signals: the first a priming signal and the second an activation signal. The priming signal serves to increase the synthesis of NLRP3 and pro-IL-1β proteins. This first signal takes place via the activation of TLR by a multitude of microbial ligands or via the activation of the TNF-α receptor. These activations will trigger the translocation of NF-κB in the nucleus and thus increase the transcription of NLRP3 and pro-IL-1β. This channel can be activated by setting the LPS on toll-like receptor 4 [30]. Consequently, we investigated whether RWE could alter the priming signal from lipopolysaccharides (LPS). To do this, Raw264.7 and J774A.1 cells were pre-incubated for 30 min with 100 μg/mL of RWE then with LPS (1 μg/mL for 5.5 h) to induce a priming end and we then measured IL-1β secretion. Secreted IL-1β in cell supernatants in both experimental conditions was then measured by ELISA. Consistent with the literature, RSV alone significantly decreased IL-1β secretion in macrophage cell lines (Figure 2a). In a manner similar to the RSV-positive control, RWE strongly decreased IL-1β production in J774A.1 and much more slightly in Raw264.7 macrophages (Figure 2a). Since LPS priming controls, at the end of its signaling cascade, pro-inflammatory IL-1β and NLRP3 transcription and finally their protein expression, we evaluated the effect of RWE on inflammasome protein expression in basal and priming conditions. Immunoblotting showed that RSV strongly downregulated NLRP3 protein expression in RAW 264.7, which was not the case in J774.1, but as previously, in the latter cells, RWE strongly reduced ASC protein expression as compared to the control when priming was implemented by the LPS (Figure 2b). We confirm that Raw264.7 macrophages did not express ASC (Figure 2c).

### 3.3. RWE Decreases IL-1β Secretion after Activation by LPS/Nigericin in Macrophages

**Figure 3:**
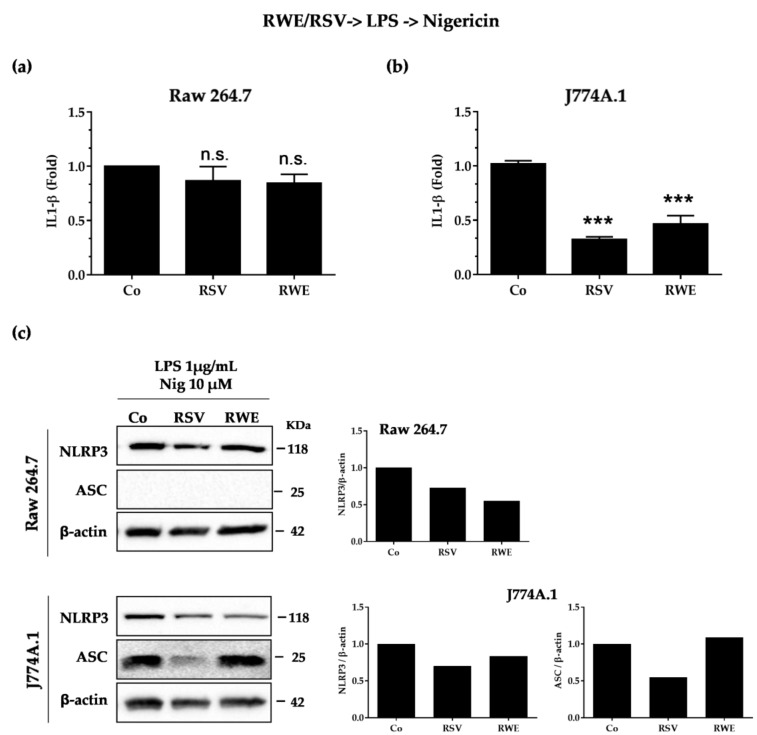
RWE and RSV curtail NLRP3 inflammasome priming and reduce IL-1β secretion, in the nigericin-mediated activation condition. Raw 264.7 (**a**) and J774A.1 (**b**) macrophages were pretreated or not with RWE (100 μg/mL) or RSV (60 μM) for 30 min, prior to NLRP3 inflammasome priming with 1 μg/mL of LPS for 5.5 h and subsequent activation by 10 μM nigericin for 30 min. Secreted IL-1β in cell supernatants was analyzed by ELISA at the end of the treatments. Data are expressed as fold changes in secreted IL-1β levels as compared to cells treated with only LPS + nigericin (Co) and are mean ± s.d. of three independent experiments. *p*-values were determined by the multiple Student *t* test. n.s.: not significant, *** *p* < 0.001. (**c**) Western-blot analysis of NLRP3 and ASC protein expression in Raw 264.7 and J774A.1 macrophages after cell treatments as described above. Left panels: representative blots from three independent experiments are shown. β-actin was used as loading control. Right panels: densitometry quantifications of representative blots. Plotted data are fold changes as compared to LPS-treated cells (Co).

After increasing the expression then the synthesis of NLRP3 and pro-IL-1β, the second signal serves to activate NLRP3, which is deubiquitinated while the adapter protein ASC is phosphorylated and ubiquitinated [31]. The permeabilization of membranes by toxins such as nigericin is one of the activation mechanisms of NLRP3 [32]. Therefore, to study the potential effect of RWE on this second signaling process, we treated J77A.1 cells with RWE (100 μg/mL) or RSV (60 μM) for 30 min before treatment with LPS for 5.5 h (LPS priming) following incubation with nigericin (10 μM for 30 min). Very surprisingly, we observed that both RWE and RSV failed to decrease the IL-1β secretion in RAW 264.7 (Figure 3a), which is not the case in J774.1 cells where RWE and RSV completely blocked the production of IL-1β induced by nigericin (Figure 3b). Concerning the expression of protein involved in the inflammasome complex, we observed that RSV and RWE decreased NLRP3 protein expression in RAW 264.7, but only RSV was able to decrease ASC protein expression in J774.1 after nigericin stimulation (Figure 3c). These results differ from previous results wherein priming conditions, RSV and RWE were both able to decrease, in J774.1, the expression of two key proteins NLRP3 and ASC, which was not the case in the second signaling process. It appears from these results that the very large decrease in IL-1β by RWE in J774.1 macrophages is associated with a strong decrease of NLRP3 protein expression.

### 3.4. Crucial Choice of Activators for RWE and RSV Effect on IL-1β Secretion in Macrophages after LPS Priming

**Figure 4:**
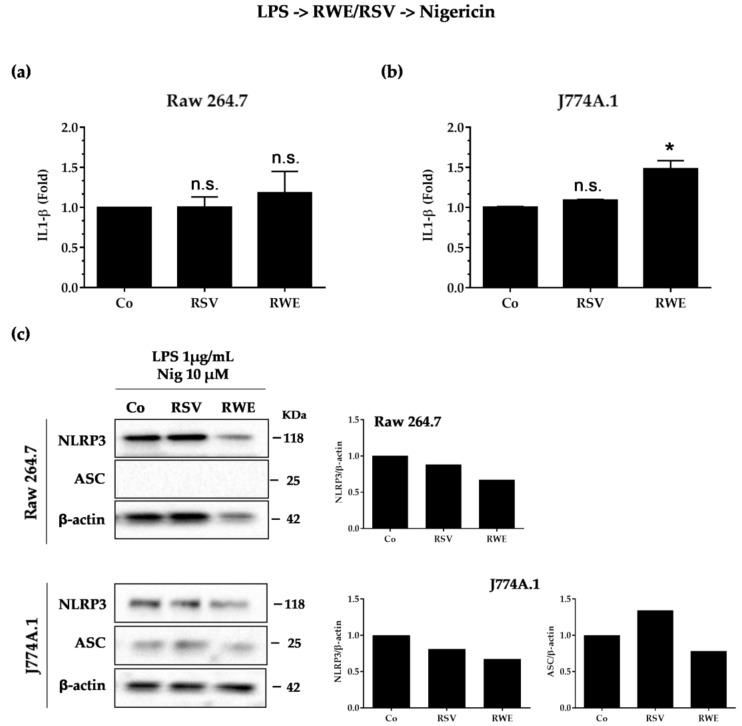
RWE and RSV failed to block IL-1β secretion when added after the NLRP3 priming step. Raw 264.7 (**a**) and J774A.1 (**b**) macrophages were exposed to 1 μg/mL of LPS for 5.5 h, then treated or not with RWE (100 μg/mL) or RSV (60 μM) for 30 min and finally exposed for an additional 30 min to 10 μM nigericin. Secreted IL-1β in cell supernatants was analyzed by ELISA at the end of the treatments. Data are expressed as fold changes in secreted IL-1β levels as compared to cells treated only with LPS + nigericin (Co) and are mean ± s.d. of three independent experiments. *p*-values were determined by the multiple Student *t* test. * *p* < 0.05, n.s.: not significant. (**c**) Western blot analysis of NLRP3 and ASC protein expression in Raw 264.7 and J774A.1 macrophages after cell treatments as described above. Left panels: representative blots from three independent experiments are shown. β-actin was used as loading control. Right panels: quantifications by densitometry of representative blots. Plotted data are fold changes as compared to LPS-treated cells (Co).

Subsequently, we evaluated the ability of RWE to interfere with the second signal “the activation”. We first treated macrophages with LPS (1 μg/mL for 5.5 h) and then treated or not with RWE (100 μg/mL) or RSV (60 μM) for 30 min and finally exposed for an additional 30 min to 10 μM nigericin. We surprisingly observed that both RSV and RWE failed to decrease IL-1β production as previously observed (Figure 4a), even though only RWE decreased NLRP3 and ASC protein expression in both types of macrophages as compared to RSV, which presented no effects (Figure 4c).

**Figure 5:**
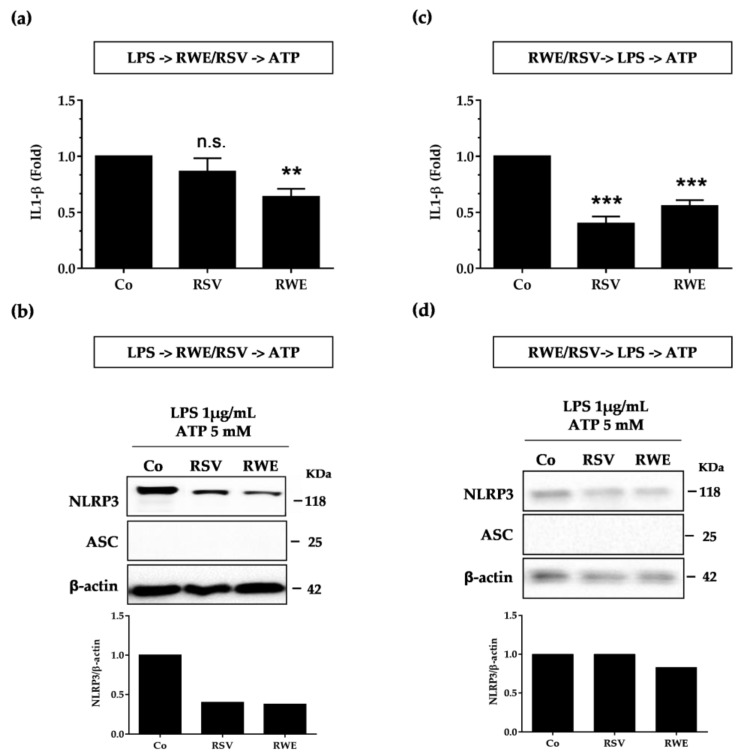
RWE prevents both priming and activation from ATP in macrophages. (**a**) LPS -> RWE/RSV -> ATP sequence: Raw 264.7 cells were exposed to 1 μg/mL of LPS for 5.5 h, then treated or not with RWE (100 μg/mL) or RSV (60 μM) for 30 min and finally exposed for an additional 30 min to 5 mM ATP. Secreted IL-1β in cell supernatants was analyzed by ELISA at the end of the treatments. Data are expressed as fold changes in secreted IL-1β levels as compared to cells treated only with LPS + ATP (Co) and are mean ± s.d. of three independent experiments. *p*-values were determined by the multiple Student *t* test. ** *p* < 0.01, *** *p* < 0.001, n.s.: not significant. (**b**) Western blot analysis of NLRP3 and ASC protein expression in Raw 264.7 macrophages after cell treatments as described in (**a**). Representative blots from three independent experiments are shown. β-actin was used as loading control. Histograms: quantifications by densitometry of representative blots. Plotted data are fold changes as compared to LPS+ATP-treated cells (Co). (**c**) RWE/RSV-> LPS -> ATP sequence: Raw 264.7 cells were pretreated or not with RWE (100 μg/mL) or RSV (60 μM) for 30 min, prior to NLRP3 inflammasome priming with 1 μg/mL of LPS for 5.5 h and subsequent activation by 5 mM nigericin for 30 min. Secreted IL-1β in cell supernatants was analyzed by ELISA at the end of the treatments. Data are expressed as fold changes in secreted IL-1β levels as compared to cells treated only with LPS + ATP (Co) and are mean ± s.d. of three independent experiments. *p*-values were determined by the multiple Student *t* test. ** *p* < 0.01, *** *p* < 0.001. (**d**) Western blot analysis of NLRP3 and ASC protein expression in Raw 264.7 macrophages after cell treatments as described in (**c**). Representative blots from three independent experiments are shown. β-actin was used as loading control. Histograms: densitometry quantifications of representative blots. Plotted data are fold changes as compared to LPS+ATP-treated cells (Co).

**Figure 6:**
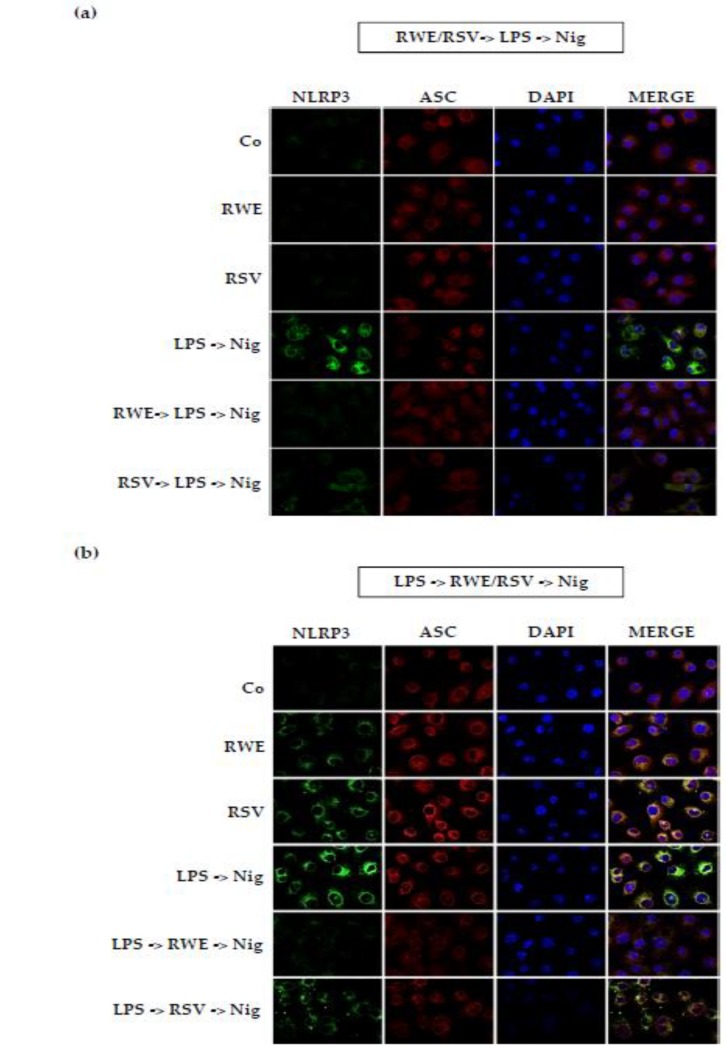
RWE and RSV decrease NLRP3 and ASC colocalization in both priming and activation conditions. (**a**) RSV and RWE inhibit NLRP3 and ASC formation in priming sequence RWE/RSV-> LPS -> nigericin. J774A.1 macrophages were exposed to 1 μg/mL of LPS for 5.5 h, then treated or not with RWE (100 μg/mL) or RSV (60 μM) for 30 min and finally exposed for an additional 30 min to 10 μM nigericin. (**b**) RSV and RWE inhibit NLRP3 and ASC formation in activating sequence LPS -> RWE/RSV -> nigericin. J774A.1 macrophage cells were exposed to 1 μg/mL of LPS for 5.5 h, then treated or not with RWE (100 μg/mL) or RSV (60 μM) for 30 min and finally exposed for an additional 30 min to 10 μM nigericin. In (**a**) and (**b**) after treatments, cells were fixed and then immunostained for NLRP3 and ASC proteins using Alexa Fluor^®^ 488 (green staining) and Alexa Fluor^®^ 568 (red staining) as secondary antibodies, respectively. Cell nuclei were counterstained with DAPI. Representative confocal images of the staining from three independent experiments are shown (×40 magnification).

Given this very surprising lack of an effect of RSV, whereas Chang et al. previously described its ability to block IL-1β secretion when macrophages were pretreated with the LPS before RSV treatment and ATP stimulation [22], we decided to change NLRP3 inflammasome activators. We consequently conducted the same experiment as before by replacing the nigericin with ATP. In this second experiment, macrophages were treated with LPS (1 μg/mL for 5.5 h) and then treated or not with RWE (100 μg/mL) or RSV (60 μM) for 30 min and finally exposed for an additional 30 min to 5 mM ATP. We then observed that RSV was able to decrease NLRP3 protein expression without modulating IL-1β secretion while RWE, in these conditions, both decreased IL-1β production and NRLP3 protein (Figure 5a,b), suggesting that RWE inhibits the ATP-mediated activation signal of the NLRP3 inflammasome. The ability of RWE to inhibit IL-1β production was also found when RAW 264.7 macrophages were pretreated with RSV prior to activation of the NLRP3 inflammasome by LPS/ATP. In accordance with the first results shown in Figure 3, RWE strongly reduced IL-1β production and NLRP3 protein expression (Figure 5c,d). Consequently, RWE was able to prevent priming of NLRP3 inflammasome from LPS in macrophages whether the activator ATP or nigericin was used. These molecular observations were reinforced *in cellulo*, where immunofluorescence imaging revealed that RWE is well able to decrease NLRP3 and ASC expression, as shown by the decrease of fluorescence of these proteins but also of their colocalization when macrophages are pretreated with RWE (Figure 6a). The same observations are highlighted when macrophages were pretreated with LPS before treatment with RWE (Figure 6b).

## 4. Discussion and Conclusions

Inflammation can be initially defined as a set of physiological defense reactions put in place by the body and more particularly by the cells of innate immunity. These cells play a role in the first line of defense following various injuries caused by infectious agents, chemical substances, physical agents, or even post-traumatic tissue lesions. In contrast, inflammation has been described as an initiator event of major diseases with significant impact in terms of public health such as cardiovascular diseases, autoimmune diseases, eye diseases, age-related diseases, and more particularly neurodegenerative diseases and the occurrence and development of cancers. A preventive action to reduce the key processes leading to inflammation could, therefore, be very interesting in order to reduce the associated pathologies.

The inflammatory response is often linked to the action of intracellular multiprotein complexes called inflammasomes. These complexes generally consist of a receptor and an adapter allowing the recruitment and activation of pro-inflammatory caspases as well as the maturation and secretion of pro-inflammatory interleukins such as IL-1 [33]. Among these inflammation-inducing complexes, the NLRP3 inflammasome is the most widely studied complex and is responsible for the production of interleukin-1. The activation of this NLRP3 complex results mainly from two signals: signal 1 called priming and signal 2 called activation of NLRP3. The priming step of the NLRP3 inflammasome corresponds to the pretreatment of the macrophages with bacterial compounds such as LPS, capable of greatly increasing its activation [34], which could result from an interaction of NLRP3 with the IRAK1 protein [35]. In this sequence, in a basal condition, without pretreatment by LPS, we first showed that RWE and RSV were able to decrease IL-1β secretion in the two macrophage types associated with strong decreases of NLRP3 and ASC proteins in J774.1 macrophages. When NLRP3 is activated by LPS, we observed that pretreatment with RWE and RSV was able to attenuate IL-1β secretion, more specifically in J774.1, and in all types the key protein expression of NLRP3 and ASC. These first observations show the capacities of RWE to decrease the pro-inflammatory cytokine IL-1β and the NLRP3 complex with a better effect than RSV alone, particularly to downregulate ASC protein expression (Figure 2).

In the second step, we investigated the ability of RWE to modulate the second signal, activation. Activation of NLRP3 will result in aberrant mitochondrial homeostasis, leading to acetylated α-tubulin accumulation. This α-tubulin is responsible for the transport of mitochondria and will allow contact between ASC and NLRP3 [36]. Once formed, the NLRP3 inflammasome causes cleavage of pro-caspase-1 in active caspase-1, which in turn will cleave pro-IL-1β to IL-1β, which will then be excreted [37]. The mechanisms leading to the activation of this are not yet clearly defined, but it seems that some compounds, such as ATP [38] and nigericin [32], could activate the NLRP3 inflammasome. We thus pretreated macrophages with RWE or RSV and then treated them with LPS for the priming signal and with nigericin for the activation signal. It appears that only in J774.1 were RSV and RWE able to decrease IL-1β secretion and NLRP3 inflammasome significantly as compared to RAW macrophages (Figure 3). Very surprisingly, when the activator is changed and substituted by another such as ATP, we observed for the same treatment sequence that RWE and RSV are then able to greatly reduce the production of IL-1β and the expression of NLRP3 proteins (Figure 5c,d). This is much more complicated when studying the effect of RWE and RSV on the second signal and not on the first and second signal sequence. Indeed, we observed that RWE decreased NLRP3 and ASC protein expression, which is not the case for RSV (Figure 4C), but in all cases, both RWE and RSV failed to decrease IL-1β production, when the activator is nigericin. This is not the case when the activator is ATP, where RWE strongly decreases IL-1β production and NLRP3 protein expression (Figure 5a,b). The discrepancies observed between the two results could result from a different mechanism with the two activators. Indeed, it seems that extracellular ATP binds to its P2X7 receptor and then stimulates intracellular proton efflux. The result is the creation of Pannexin-1 membrane protons that may allow extracellular components to enter the cytosol and activate NLRP3 [38]. Some studies have proven that intracellular ATP is required for cellular functions such as the biosynthesis of pro-inflammatory cytokines and the maintenance of mitochondrial membrane potential. Therefore, a decrease in these levels might be a potent signal that activates the NLRP3 inflammasome [39]. According to other studies, the variation in intracellular potassium would be sufficient to induce the activation of NLRP3. In this way, some bacterial toxins such as nigericin leading the formation of ports in the plasma membrane also cause potassium efflux [32]. Further investigations should specify the actors involved in these two distinct mechanisms where one of the activators, namely ATP, does not require the ASC adapter protein for activation and secretion of IL-1β.

In addition to highlighting the effects of RWE on the activation of the inflammasome for the first time, we showed that a complex mixture of polyphenols can exhibit biological effects superior to a single molecule such as RSV. These new findings on inflammation support those obtained in the context of colorectal carcinogenesis where we previously demonstrated that an extract enriched in polyphenols could exhibit synergistic antiproliferative and antineoplastic effects in models of chemo-induced cancers in mice [18]. Qualitative and quantitative polyphenolic compositions are crucial for the determination of biological effects, especially on the occurrence of synergistic effects and additives seen for some antagonists’ effects. We previously showed that resveratrol and quercetin combined exhibited a synergistic effect inducing apoptosis, antiproliferative effects, and cell cycle disruption compared to the compounds alone in colon cancer cells. Interestingly, in this same study, the combination of resveratrol+catechin or resveratrol+catechin+quercetin produced only an additive effect on the inhibition of tumor cell proliferation or on cell cycle arrest, and catechin+quercetin did not induce a synergistic effect on apoptosis [18]. In the present study, we observed that RWE presented a greater effect than RSV in various conditions, i.e., decreasing ASC protein expression in basal conditions, decreasing both ASC and NLRP3 protein expression and IL-1β secretion after priming by LPS, and interfering with the second signal to decrease ASC/NLRP3 protein expression and IL-1β secretion when ATP is used as an activator. These better effects can again result from synergies between the various compounds present in the polyphenol red wine extract. Indeed, the analysis of polyphenolic composition reveals the presence of resveratrol, catechin/epicatechin, and quercetin derivatives. These compounds are particularly important since separately they have been shown to exert an action on inflammation. For example, catechin was able to inhibit MSU-IL-1β secretion and NLRP3 inflammasome activation in THP-1 [40]. Similarly, quercetin was able to inhibit NLRP3 activation by interfering with ASC oligomerization and prevented IL-1β secretion in macrophages [41]. A possible mechanism of synergism could result from quercetin. We previously showed that a combination of resveratrol+quercetin increases resveratrol uptake by colon carcinoma cells, and in combination with RWE this combination increases uptake of resveratrol [18]. Similarly, the presence of quercetin in RWE could increase the uptake of RSV as well as others in macrophages to potentiate their effects against inflammasome activation.

In conclusion, many studies have highlighted the value of a polyphenol of the grapevine and wine, resveratrol, to reduce the production of proinflammatory cytokines, especially in the context of cardiovascular pathologies and cancers. However, resveratrol is not the only polyphenol of interest: other polyphenols of nutritional interest could play an important role, including those present in wine. We point out that depending on the activator used, ATP or nigericin, the effects observed can sometimes differ. In view of these results, experiments will have to be conducted to study if the modulation of inflammation by polyphenols of the extract can also be related to a modulation of the immune system, a major player in the production and maintenance of the cytokine balance.

## Figures and Tables

**Table 1:** Composition of the red wine extract (RWE) obtained from the 2012 Santenay 1er cru Les Gravières.

	mg/L of Wine	mg/L RWE
**Phenolic acids**		
Gallic acid	21	5.04
Caftaric acid	53	12.74
Coutaric acid	20	4.80
Caffeic acid	10	2.40
**Stilbenes**		
Piceid	1	0.24
Resveratrol	7	1.68
**Anthocyanidins**		
Delphinidin derivatives	15	3.60
Petunidin derivatives	10	2.40
Peonidin derivatives	14	3.36
Malvidin derivatives	78	18.75
**Catechins**		
Catechin	31	7.45
Epicatechin	8	1.92
Procyanidin dimers	42	10.09
**Flavonols**		
Quercetin derivatives	4	0.96
